# A Comparative Study of Effects of Biodegradable and Non-biodegradable Microplastics on the Growth and Development of Black Soldier Fly Larvae (*Hermetia illucens*)

**DOI:** 10.1007/s12649-023-02296-0

**Published:** 2023-10-21

**Authors:** Carina D. Heussler, Isabel L. Dittmann, Bernhard Egger, Sabine Robra, Thomas Klammsteiner

**Affiliations:** 1 Department of Ecology, Universität Innsbruck, 6020 Innsbruck, Austria; 2 Department of Zoology, Universität Innsbruck, 6020 Innsbruck, Austria; 3 Department of Waste Treatment and Resource Management, Universität Innsbruck, 6020 Innsbruck, Austria; 4 Gopa Worldwide Consultants, 61348 Bad Homburg vor der Höhe, Germany; 5 Department of Microbiology, Universität Innsbruck, 6020 Innsbruck, Austria

**Keywords:** Microplastic mismanagement, Insect-based bioconversion, Histology, Circular economy, Insect frass, Organic fertilizer

## Abstract

**Purpose:**

This study aimed to investigate the digestion process of biodegradable and non-biodegradable microplastics (MPs) within black soldier fly larvae (BSFL) and assess their impact on larval growth and development. The goal was to understand the fate of MPs within BSFL, considering their potential for waste conversion polluted with MPs.

**Methods:**

BSFL were exposed to two types of MPs, and their growth, development, potential accumulation and excretion of MPs were monitored.

**Results:**

The findings revealed that the MPs accumulated solely in the larval gut and had no adverse effects on the growth and development of BSFL. Larvae efficiently excreted MPs before reaching the pupation stage.

**Conclusion:**

This research emphasizes the potential of BSFL as a bioconversion agent for organic waste, even in the presence of MPs. The effective excretion of MPs by BSFL before pupation suggests their ability to mitigate potential harm caused by MP accumulation. The fact that BSFL may excrete MPs before pupation would contribute to their safe use as animal feedstock. A careful evaluation of the effects of using BSFL reared on contaminated substrates especially containing visually non-detectable residuals like nanoplastics, chemicals or toxic metals and further examination of the broader implications for waste management and sustainable livestock farming remains important.

**Graphical Abstract:**

Experimental design outlining the workflow for the analyses used to investigate the effect of two types of microplastics, polyamide (PA), and polylactic acid (PLA), on growth and development of black soldier fly larvae.

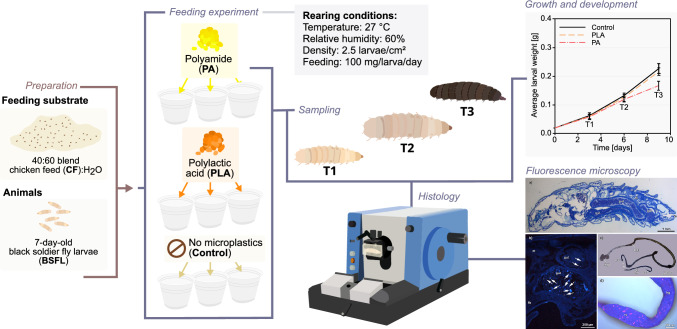

## Statement of Novelty

This study provides novel insights into the efficient digestion and excretion of microplastics (MPs) by black soldier fly larvae (BSFL), demonstrating their potential as a safe bioconversion agent for organic waste contaminated with MPs. The BSFL's capacity to excrete MPs before pupation showcases their potential in reducing the risk of MP accumulation, opening new possibilities for sustainable waste management and livestock farming.

## Introduction

Since the 1950s, plastic has become a global everyday material. Its production has grown from 1.5 million tons (1950) to 368 million metric tons (2019) and it is estimated that the production will grow to approximately 1.8 billion tons by 2050 [1, 2]. Most plastic products were and still are intended for single-use, leading to a massive waste management problem and ecological pressure. Mostly, plastic waste is not properly discarded and ends up in landfills, where about 20–42% is stored on land [3]. Due to its high environmental persistence, it biodegrades very slowly (up to 500 years) and about 10% of all plastic flows enter streams, rivers, and oceans, thus endangering aquatic life [3]. It has been predicted that by 2050 there will be more plastic mass than fish mass in the ocean [4]. Due to physicochemical and biological forces, plastics entering the environment break up into macro- and microplastics [5]. Although definitions vary, macroplastics are generally considered to start at dimensions of > 25 mm, while microplastics (MP) are classified as particles 1–5 mm in size [6].

Two main sources have been reported for the production of MPs: one source is the mismanagement of plastic waste (e.g. single-use plastic), and the second source is MPs originating from microbeads from cosmetics, fibres from clothing, and tiny granules from industry [7–9]. Consequently, MPs are now present in most, if not all, ecosystems, altering their biophysical, microbiological, and chemical characteristics [10]. Furthermore, it has been shown that MPs can also enter plant cells via endocytosis [10, 11], or are consumed by animals, thus endangering also human health via food-chain toxicity [9, 12]. As a consequence, they also accumulate in agricultural and food waste.

The decomposition of organic waste (e.g., sewage sludge, agricultural and food waste, the organic fraction of municipal household wastes) using black soldier fly larvae (BSFL) has gained interest in the last decades, as it is generally considered safe, sustainable, and cost-efficient [13]. Furthermore, BSFL and their rearing residues (frass) can be further processed into livestock feed and fertiliser, respectively, thus contributing to a circular economy [14, 15].

While MPs were observed to alter the survival, growth, metabolism, and even behaviour of many insect species [16, 17], recent studies indicate that MPs (polyethylene, polypropylene, polyvinyl chloride, and polystyrene) do not negatively affect growth and performance of BSFL [16, 18]. Xu et al. [19] showed that the presence of MPs (polyvinyl chloride) can alter the gut microbiome, with negative effects on the digestion of the organic matter. Therefore, this study aimed to address the following questions: (i) Do BSFL ingest MPs? (ii) If so, do the particles accumulate in their guts or are they excreted? (iii) Do MPs affect larval growth? Furthermore, we aimed to assess the different effects of a non-biodegradable MP (polyamide, PA) and a biodegradable MP (polylactic acid, PLA). PLA has a wide application in several sectors of everyday life. Even though it is a biodegradable polyester, it only degrades under specific conditions, e.g. sufficient oxygen concentrations and high temperature (58–80 °C), high humidity (< 60% RH), and the presence of microorganisms [20]. PA is ubiquitous in household commodities, clothes, and industry, and its residues often end up in the environment [21]. Considering the diverse applications of BSF in the organic waste management sector, it is reasonable to assume that BSF will come into contact with various types of microplastics. Consequently, it is essential to analyse the impact and processing of microplastics within the BSF ecosystem.

To this end, we determined larval growth and survival, pupal development, and pupation rate of BSFL that were fed diets spiked with non-biodegradable and biodegradable MPs. Furthermore, we observed how MPs were processed in the larvae via fluorescence microscopy.

## Material & Methods

### Microplastics and Feed Preparation

UV-fluorescent plastic items were obtained from an online store specialising in party equipment (https://www.schwarzlicht.de). UV-reflecting orange PA knitting wool and yellow PLA filament for 3D printers were used to represent biodegradable plastics. The materials were cut into small pieces and repeatedly ground in a CryoMill (Retsch, 42,781 Haan, Germany) until the desired particle size was achieved (< 150 μm).

A concentration of 0.22% (w/w) based on Romano and Fischer [18] for each of the MPs (PA and PLA) was added to dry ground chicken feed (CF; Grünes Legekorn Premium, Unser Lagerhaus WHG, Austria) and subsequently mixed with tap water at a ratio of 40:60 (w/v). Additionally, a control treatment containing only a freshly prepared mixture of CF and water (40:60, w/v) was included, which had been fed to several generations of BSF before.

### Insects and Experimental Design

The BSFL were obtained from a black soldier fly lab colony kept in a climate chamber at the Department of Ecology (Universität Innsbruck, Innsbruck, Austria) under stable environmental conditions of 27 °C and 60% relative humidity. The neonate BSFL were nursed in black plastic boxes (180 × 120 × 80 mm) and fed with an *ad libitum* amount of a 40:60 mixture of CF and tap water for 7 days. For each replicate, 96 7-day-old BSFL were manually counted and transferred to separate plastic cups (Ø = 7 cm, A = 38.5 cm^2^; n = 3) at a density of 2.5 larvae cm^2^ [22]. The larvae were fed in a ratio of 100 mg/d/larva. Feeding occurred every 3 days and the biomass of the larvae was determined at T1 (3-day feeding), T2 (6-day feeding), and T3 (9-day feeding), right before the fresh feed was added, by weighing 10 randomly selected BSFL per replicate using an analytical balance (Mettler Analytical Balance AE 166 Delta Range, Mettler-Toledo Ltd., Columbus OH, USA; accuracy of scale display 0.001 g). Additionally, 7 BSFL from each replicate were sampled for gut analysis and stored at − 20 °C. The feeding was terminated after 9 days (at a larval age of 16 days) when pupation had started in all replicates. After determining the biomass of the BSF pupae (BSFP), samples thereof were stored at − 20 °C for pupal cell pulp analysis.

Mortality, pupation-, and growth rate were calculated as follows:

1 $$\begin{gathered} {\text{Mortality rate}}:~~~\left( {ndL*100} \right)/niL \hfill \\ \quad \quad \quad \quad \quad \quad \quad \quad ndL = niL{-}\left( {neL + nP} \right) \hfill \\ \end{gathered}$$

2 $${\text{Pupation}}\,{\text{rate}}:~~\left( {100*nP} \right)/(neL + nP)$$

niL = Number of larvae input, neL = Number of larvae at the end of the experiment, nP = Number of pupae, ndL = Number of dead larvae.

3 $${\text{Growth}}\,{\text{rate}}:~\,\left( {weL{-}wiL} \right)/T3$$

weL = Weight of larvae at the end of the experiment, wiL = Weight of larvae at the time of input, T3 = 9 days after start of feeding.

### Histology

Histological examinations of BSFL whole-mount specimens were performed. One group of the exanimated BSFL were priorly reared on CF supplemented with 0.22% of polyamide (PA), and a control group reared on CF without MP particles. Larvae were relaxed for 10–15 min at 4 °C and fixed in 10% formaldehyde in 0.2 M Soerensen’s phosphate buffer. To infuse the specimens, the epidermis was incised. Afterwards, larvae were embedded in Technovid 9100 (Kulzer, Wehrheim, Germany) following the enclosed manufacturer’s protocol. Specimens were sectioned at 3 μm using a Reichert-Jung Autocut 2040 microtome (Leica Biosystems, Vienna, Austria) and dried over 48 h at about 60–70 °C. Afterwards, specimens were stained with methylene blue [23], for 1 min.

### Gut and Pupal Cell Pulp Preparation

The BSFL and BSFP were thawed at room temperature. The guts were extracted by pulling out the anus using sterile forceps. To collect the pupal cell pulp, the pupae were cut along both lateral sides with sterile scissors and the cell pulp was scraped out with a sterile spatula. The guts and cell pulps were each placed on a microscope slide and covered with distilled water and a coverslip for better visualization.

### Fluorescence Microscopy and Documentation

Frozen BSFL and BSFP were captured with a Leica MZ16F stereomicroscope equipped with a Leica DFC450 C digital camera (Leica Microsystems Heerbrugg, Switzerland). Histologically prepared specimens, as well as feeding substrate, PLA and PA particles, and gut content, were analysed and documented with a Leica DM 5000B compound microscope equipped with a Leica DFC 490 digital camera. MPs were excited at a wavelength of 359 nm (DAPI). Image processing was performed with Adobe Photoshop 7.

### Statistical Analysis

Analysis of variance (one-way ANOVA) and a Bonferroni correction was performed in SPSS v26 (IBM, Armonk, NY, USA) for each treatment. The treatment (Control, PLA, PA) was used as the main effect. The data of larval and pupal biomass, development time, and mortality were the response variables. Data were visualized using Excel 365 Office 2021 (Microsoft Corporation, Redmond, WA, USA) and Sigmaplot v.14.5 (Inpixon, Düsseldorf, Germany).

## Results and Discussion

### Larval Development

In this study, we analysed whether or not BSFL ingested PLA and PA particles, and if so, whether these MPs accumulated in the larval tissues or were excreted. Furthermore, we analysed if the MPs affected larval growth and development, larval and pupal weight, pupation rate, mortality, and growth rate.

**Fig. 1:**
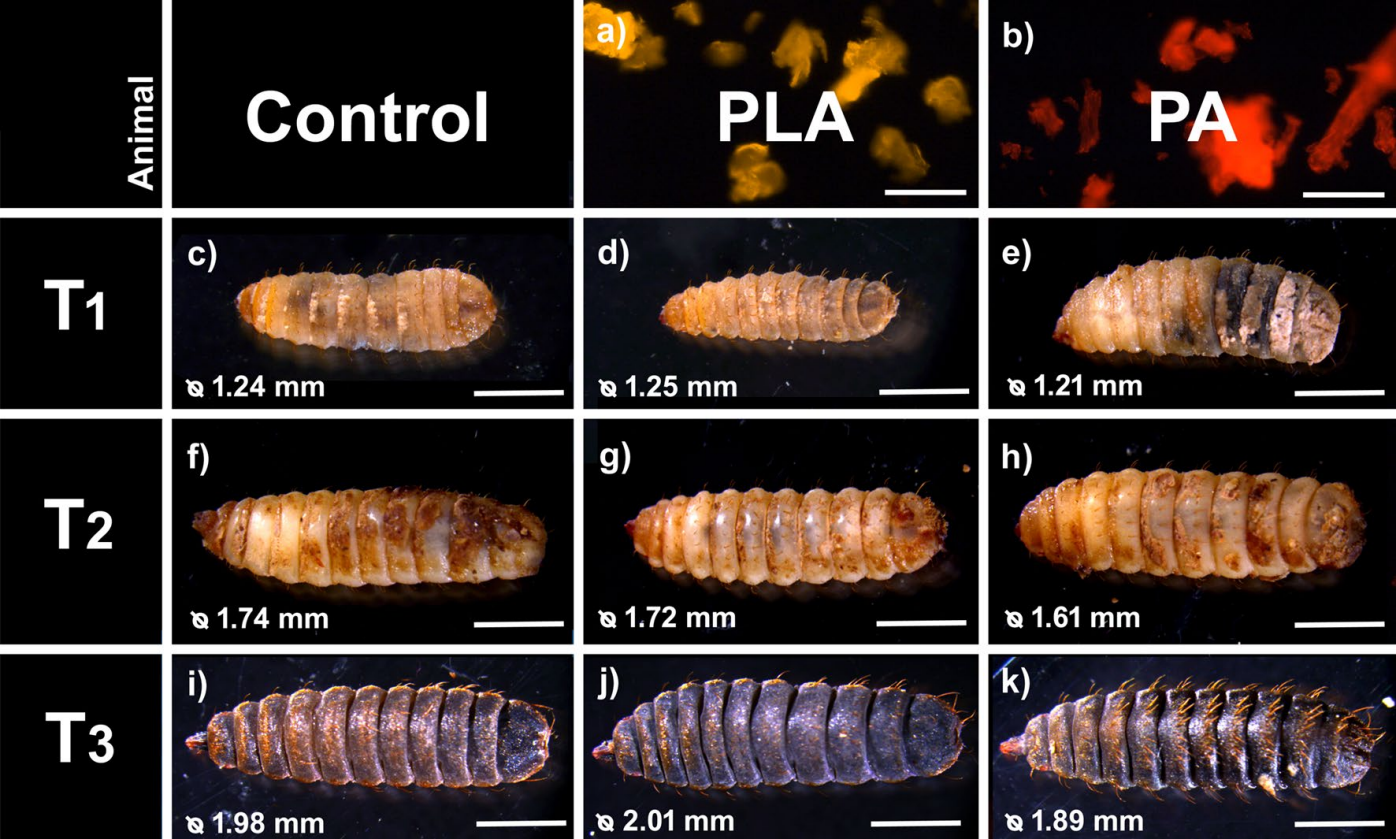
**a**, **b** Fluorescence image of pure (**a**) polylactic acid (PLA) and (**b**) polyamide (PA) particles. **a**, **b** Scale bar 100 μm. **c**–**e** T1 (3-day feeding) black soldier fly larvae (BSFL) reared on chicken feed (CF) as (**c**) a control (Control), CF supplemented with 0.22% of (**d**) PLA and (**e**) PA. **f**–**h** T2 (6-day feeding) BSFL reared on (**f**) CF as a control (Control), CF supplemented with 0.22% of (**g**) PLA and (**h**) PA. **i**–**k** T3 (9-day feeding) black soldier fly pupae reared on (**i**) CF as a control (Control), CF supplemented with 0.22% of (**j**) PLA and (**k**) PA. The value in the left corner shows the average length. **c**–**k** Scale bar 500 μm

Fluorescence microscopy showed that the prepared PLA and PA particles were similar in shape and size (Fig. 1a, b). As reported by [24], MPs often have an irregular flake shape with an irregular structure, similar to our self-produced MPs.

**Fig. 2:**
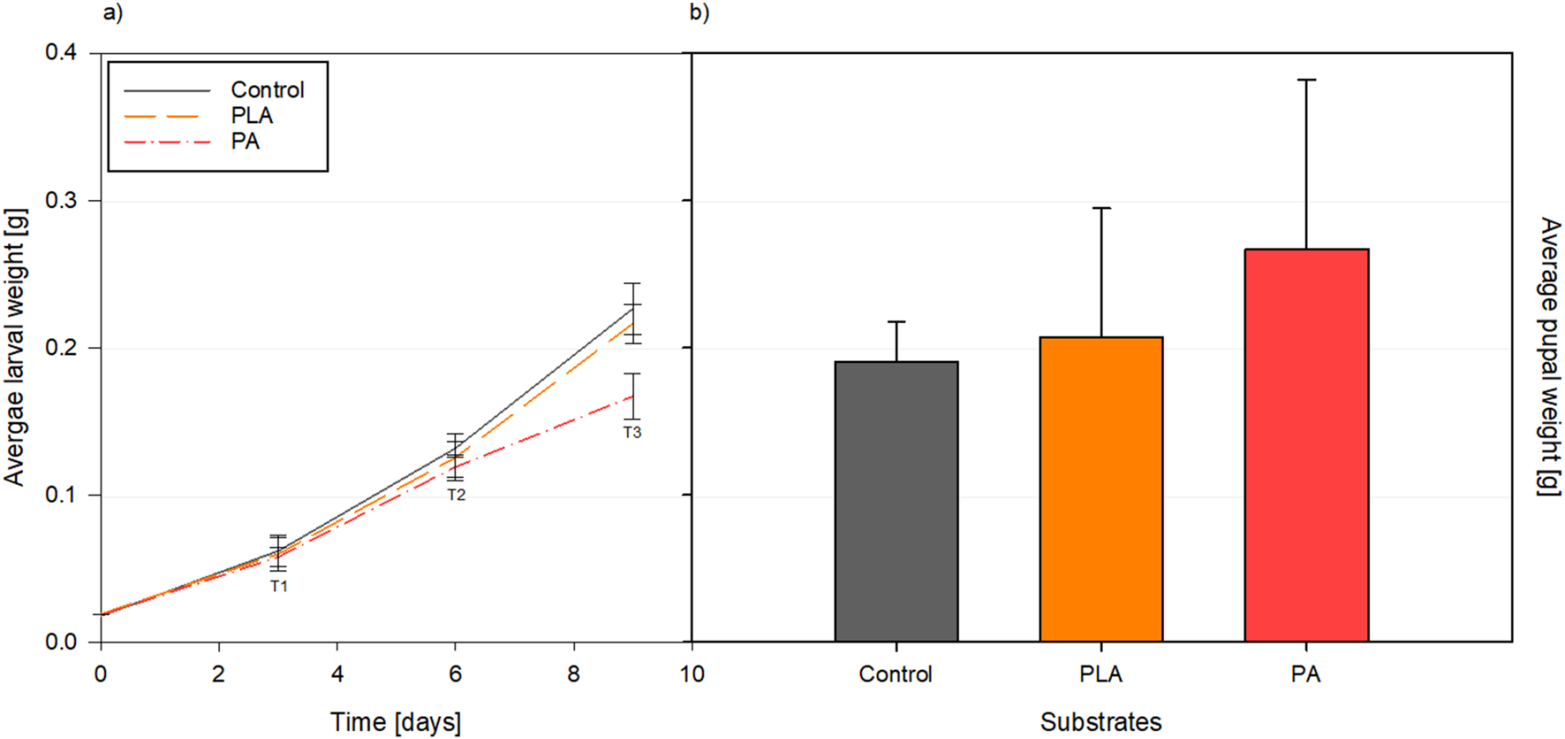
**a** Average weight (g) and standard deviation of black soldier fly larvae at T1 (3-day feeding), T2 (6-day feeding), and T3 (9-day feeding) and **b** pupae reared on chicken feed (CF) as a control (Control), CF supplemented with 0.22% of polylactic acid (PLA) and polyamide (PA). ANOVA with Bonferroni Correction was calculated for comparison between groups; however, no significant differences were found

BSFL reared on the Control and the MP treatments showed no significant difference in development, growth, pupation rate, mortality, and morphology (Figs. 1 and 2; Table 1). However, BSFL reared on PA showed a lower weight on T3, even though the pupae emerging from this diet showed the highest average weight. It is uncommon for pupae to weigh more than larvae, and further analysis is warranted to investigate the reasons behind this results. Additionally, it’s worth noting that the PA pupae showed the highest weight but also exhibited the highest standard deviation. Given that this was a small case study, the sample size may have been too low to draw definitive conclusions, and it is advisable to conduct another study with a larger sample size.BSFL reared on the Control and PLA showed a similar increase in weight throughout time, 2.3 ± 0.2 and 2.2 ± 0.2 g/day, respectively (Table 1). BSFP reared on the Control were the lightest (Fig. 2). These results are consistent with previous studies comparing polyethylene, polypropylene, polyvinyl chloride, and polystyrene [16, 18]. A study performed by Xu et al. [25], where BSFL were exposed to polyvinyl chloride and pigeon feces, indicated that the weight of BSFL was negatively influenced by the presence of MPs, though the results were not significantly different. The authors propose that the presence of microplastics (MPs) could potentially disrupt nutrient decomposition. The combination of nutrient-deficient pigeon faeces and MPs could cause an excessive strain on the larval gut, and consequently, decreased larval body weight.

Furthermore, no significant differences were found in the BSFL mortality, pupation and growth rates, and larval development times (Table 1). The average mortality was lowest for PA (9.4% ± 6.3) and highest for PLA (14.9% ± 3.7). Other studies showed similar results, where the Control was compared to MP treatment [16, 24, 26]. With 33% ± 4.3 and 32% ± 4.4 average pupation rates and 7 ± 1.7 and 7 ± 1.7 days until the first pupation, respectively, PLA and PA treatments showed similar performance. The Control resulted in a lower pupation rate (26.1% ± 1.7) and a higher development time of 9 days of feeding. A study performed by Romano and Fischer [18] showed a lower pupation rate in the presence of MPs, despite using the same MP concentrations as in our study. However, it is important to note that the type of MPs employed in their study (polypropylene) differs from the ones used in our research and could thus have a different effect on larval development. Therefore, in order to broaden the understanding of the effects of various MPs on BSFL growth, a comparative investigation encompassing multiple types of MPs and the same basic substrate should be conducted. It is also worth mentioning that a fast development time in correlation with smaller larvae can be an indicator of stress, for example, due to insufficient nutrient availability [27, 28]. Our results indicate that MPs did not cause a stress reaction during larval development, quite to the contrary: although not statistically significant, MPs seemed to have a slightly positive effect on larval biomass and development. A possible reason might be that the presence of MPs improved the growth environment of BSFL by potentially acting as a bulking agent and, thus, reducing the clumpiness of the substrate and enhancing ventilation [29, 30]. However, this effect requires further investigation, as the MP concentrations were significantly lower compared to other studies investigating the impact of MPs on insect development, but still higher than concentrations commonly found in the environment [18].

**Table 1:** Mortality-, pupation-, growth rate, and days to pupation of BSFL reared on CF (Control), CF supplemented with 0.22% of polylactic acid (PLA) and polyamide (PA). ANOVA with Bonferroni Correction was calculated for comparison between groups, however, no significant differences were found

	Control	PLA	PA
Average mortality (%)	13.5 ± 2.1	14.9 ± 3.7	9.4 ± 6.3
Average pupation rate (%)	26.1 ± 1.7	33 ± 4.3	32 ± 4.4
Average growth rate (g/day)	2.3 ± 0.2	2.2 ± 0.2	1.7 ± 0.2
Average time to the first pupation (day)	9 ± 0.0	7 ± 1.7	7 ± 1.7

### Histology and Microplastic Ingestion

**Fig. 3:**
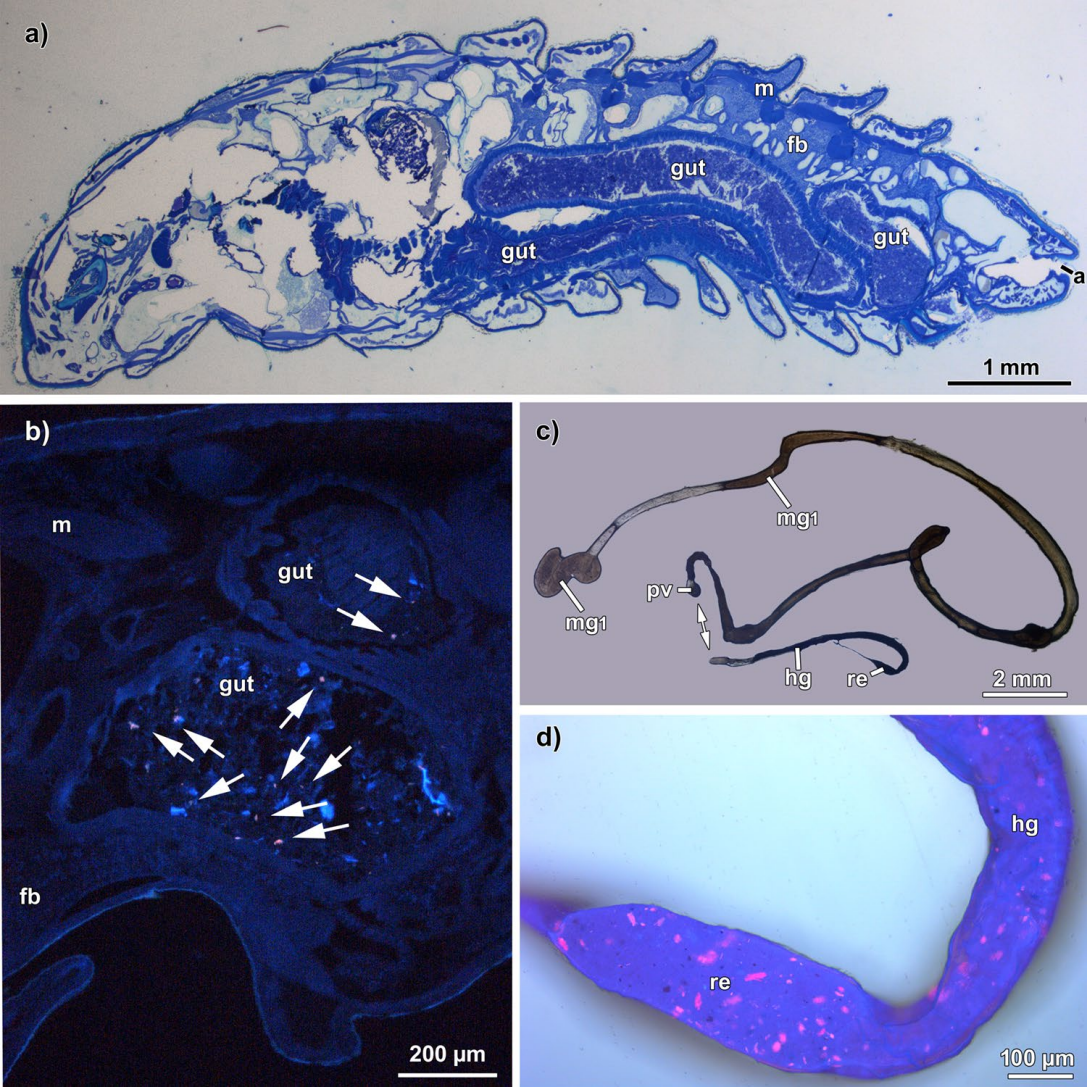
Different tissues of black soldier fly larvae at T1 (3-day feeding) reared on chicken feed mixed with polyamide (PA). **a** Sagittal section stained with methylene blue, anterior to the left. **b** Fluorescence image of different types of tissues in a sagittal section. Arrows point to microplastic particles in the gut content. **c** Overview of the removed intestinal tract. **d** Overlay image (brightfield and fluorescence) of the rectum and a part of the hindgut showing the fluorescent PA particles in the gut content. *a* anus, *fb* fatbody, *gut* gut, *hg* hindgut, *m* muscle, *mg1* mid gut region 1, *mg2* midgut region 2, *pv* proctodeal valve, *re* rectum

In a pre-experimental trial, histological examinations of BSFL reared on CF supplemented with PA particles showed that their ingestion was limited to the digestive organs (Fig. 3a, b). Therefore, we decided to focus only on the larval gut during the following experiment (Fig. 3c, d).

**Fig. 4:**
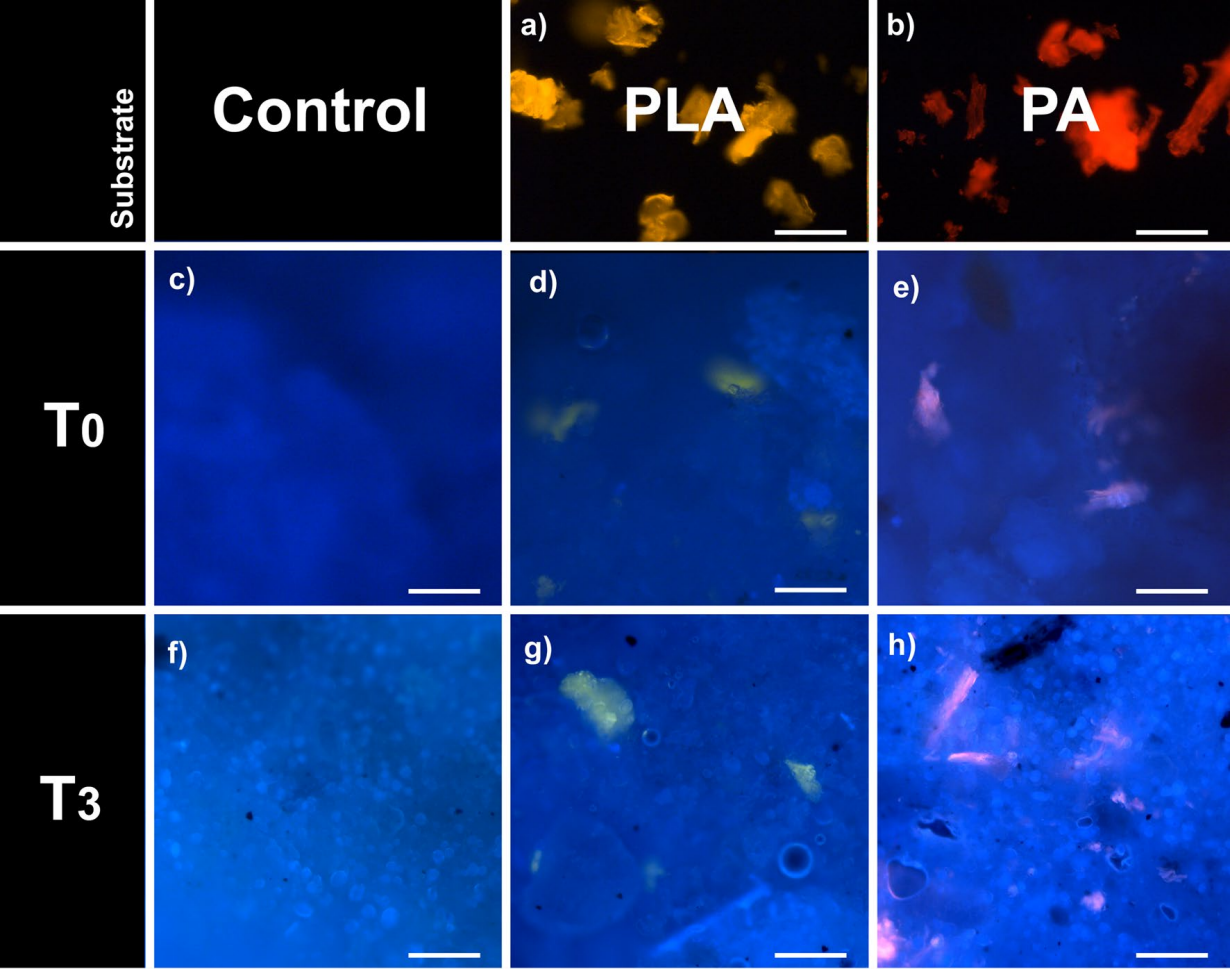
Fluorescence images of the feeding Substrate. **a** pure polylactic acid (PLA) and **b** polyamid (PA) microplastic (MP) particles. **c** Fluorescence image of the pure chicken feed (CF) without MP particles. **d**, **e** Fluorescence image of the CF mixed with (**d**) PLA or (**e**) PA before the beginning of the experiment (T0). **f** Fluorescence image of the pure CF without MP particles at the end of the experiment (T3). **g**, **h** Fluorescence image of the CF mixed with (**g**) PLA or (**h**) PA at the end of the experiment (T3). **a**–**h** Scale bar 100 μm

**Fig. 5:**
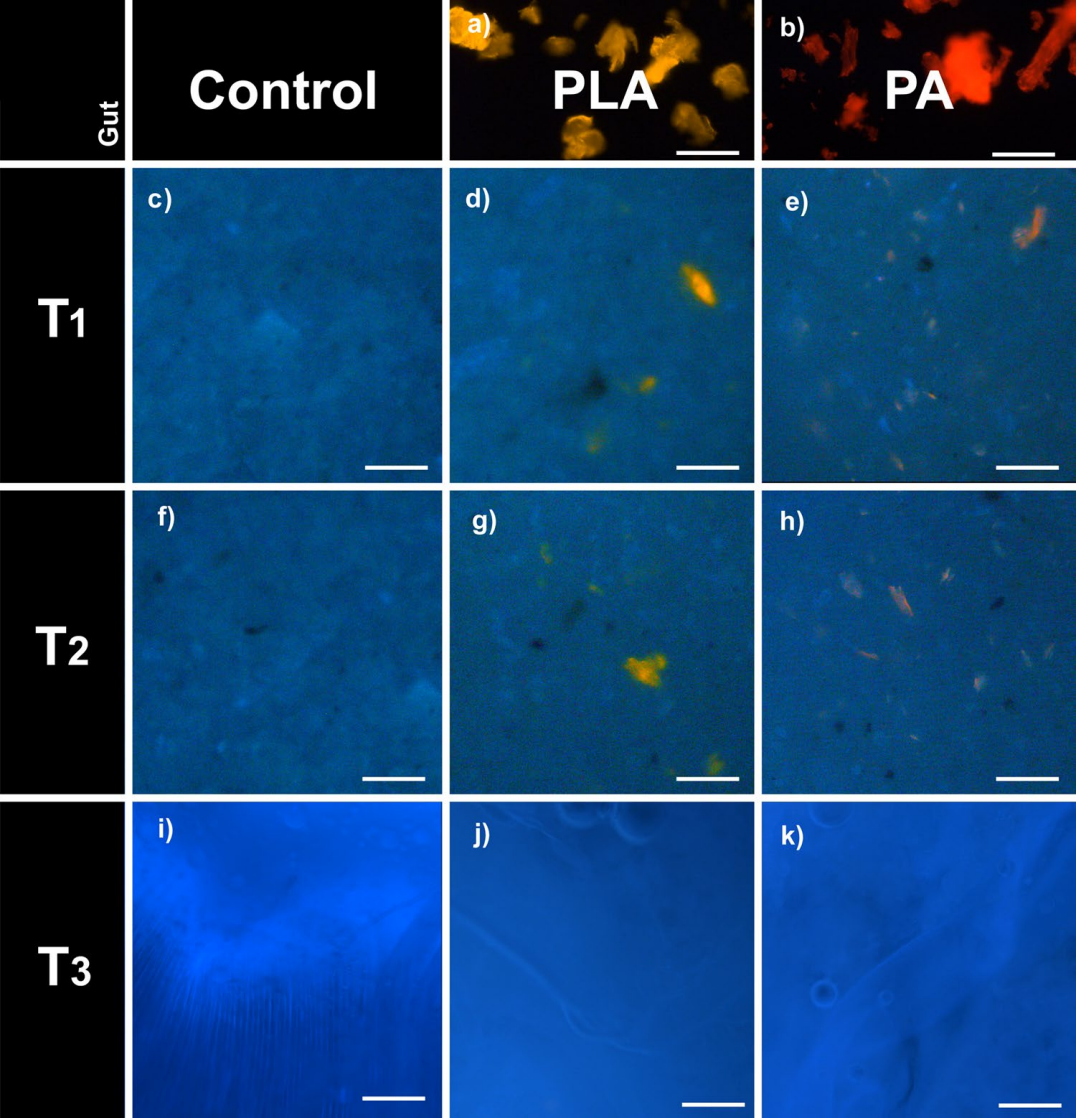
**a**, **b** Fluorescence image of pure (**a**) polylactic acid (PLA) and (**b**) polyamid (PA) microplastic (MP) particles. **c**–**e** Fluorescence image of the gut content at T1 (3-day feeding) black soldier fly larvae (BSFL) reared on (**a**) chicken feed (CF) without MP particles, (**d**) with CF mixed with PLA, (**e**) with CF mixed with PA. **f**–**h** Fluorescence image of the gut content at T2 (6-day feeding) BSFL reared on (**f**) CF without MP particles, (**g**) with CF mixed with PLA, (**h**) with CF mixed with PA. **i**–**k** Fluorescence image of the cell pulp of black soldier fly pupae at T3 (9-day feeding) (**i**) CF without MP particles, (**j**) with CF mixed with PLA, (**k**) with CF mixed with PA. **a**–**k** Scale bar 100 μm

Both types of MP (PA and PL) mixed with the feeding substrate (Fig. 4) were ingested by the larvae (Fig. 5c–h) and were detected by fluorescence microscopy in the larval gut (Figs. 3a–c and 4, 5). MP particles were detectable in all regions of the larval digestive system. However, when comparing the T1 and T2 larval gut, no MP accumulation over time is detectable. This is also in line with the study of [26], who showed that BSFL ingest and defecate MPs together with the feeding substrate. Unfortunately, the guts of BSFL at T3 were too fragile and the extraction of an entire gut and its content was not feasible. During pupation, the digestive system of BSFL undergoes a remodeling process which involves the degeneration of the corresponding larval tissue [31, 32]. No MP particles were detectable in BSFP (Fig. 5i–k). Hence, we suggest that the MP particles are excreted together with the gut content before pupation.

BSFL in the early prepupal stage, around the fifth instar, contain the highest amounts of nutrients, Therefore, this stage is best suited for the production of animal feed. Our results indicate that, at these later stages, MPs were hardly detectable or not present at all (Fig. 5j, k), which would mitigate the risk of introducing MPs into the feed chain via products derived from BSFL biomass. In addition, a starvation period before harvesting could be a possible solution to remove MPs from the larval guts [26]. While previous studies, including the findings presented here, suggest that BSFL may not readily accumulate MPs, it is advised to continue monitoring the fate of MPs during BSF rearing in a more extensive study encompassing a broader array of plastic compounds and concentrations. Additionally, the potential accumulation of other contaminants like heavy metals, pharmaceuticals, or pesticides should be considered, as they could pose a risk if ingested by BSFL and reintroduced into the food chain [24, 33].

So far, BSFL were not found to be able to degrade the MPs [24, 26]. Moreover, the role of the BSFL gut microbiome in processing ingested MPs has yet to be explored, while earlier findings have suggested that changes in the gut microbiome’s composition have no impact on its functional capacities, alongside the maintenance of a stable core microbiome [34, 35]. Comparing the substrate before (fresh) (Fig. 4c–e) and after larval digestion (frass) (Figs. 4f–h and 5c–h), no decrease in size and number of the MPs could be observed (based on visual evaluation, Figs. 4 and 5). For comparison, the yellow mealworm (*Tenebrio molitor*), can reduce ingested poly fractions by up to 65% with a 25 h gut retention time. Also, the greater wax moth (*Galleria mellonela*), can consume 84% of polyethylene in 24 h when provided only plastic substrate [36, 37]. Furthermore, LeMoine, Grove, Smith and Cassone [36] found that the putative digestive enzymes involved in lipid oxidation are higher in larvae fed a polyethylene diet, indicating that *G. mellonela* is inducing pathways to effectively metabolize the polyethylene. Since BSFL may have limited ability to degrade MPs in contaminated waste, it is important to evaluate the resulting frass carefully before using it as an organic fertilizer. Additionally, effects on the metabolic pathways of BSFL fed with MPs should be further investigated.

## Conclusion

Our study confirmed the ingestion of MPs by BSFL; however, the MPs were excreted in their original form after uptake, indicating limited, if any, degradation during the digestion process. It is important to note that the results were only observed through visualization techniques, and therefore, they must be interpreted with caution. Consistent with prior research, we found that the presence of biodegradable and non-biodegradable MPs had no adverse effects on BSFL growth and development. This demonstrates the potential of the BSF as an effective insect species for organic waste conversion, even if this waste is contaminated with MPs. However, it is important to carefully evaluate the effects of using BSFL reared on MP-contaminated substrates. Moreover, since the digested MPs can accumulate in the rearing residues, it is crucial to consider the implications of using these residues as organic fertilizer, as MPs may be introduced into the soil and food system.

## Data Availability

Enquiries about data availability should be directed to the authors.

## References

[1] Peñate, C.O.: Microplastics in drinking water, food, and everyday products. Environmental Health Association of Québec (ASEQ-EHAQ) (2021). https://aseq-ehaq.ca/wp-content/uploads/2021/08/Microplastics1-ENpdf.pdf

[2] Zheng J, Suh S (2019). Strategies to reduce the global carbon footprint of plastics. Nat. Clim. Change.

[3] Awoyera PO, Adesina A (2020). Plastic wastes to construction products: status, limitations and future perspective. Case Stud. Constr. Mater..

[4] Jennings FJ, Allen MW, Le Vu Phuong T (2021). More plastic than fish: partisan responses to an advocacy video opposing single-use plastics. Environ. Commun..

[5] Zhou Y (2023). Microplastics as an underestimated emerging contaminant in solid organic waste and their biological products: occurrence, fate and ecological risks. J. Haz. Mater..

[6] Lee J (2013). Relationships among the abundances of plastic debris in different size classes on beaches in South Korea. Mar. Pollut. Bull..

[7] Hidalgo-Ruz V, Gutow L, Thompson RC, Thiel M (2012). Microplastics in the marine environment: a review of the methods used for identification and quantification. Environ. Sci. Technol..

[8] Horton AA, Walton A, Spurgeon DJ, Lahive E, Svendsen C (2017). Microplastics in freshwater and terrestrial environments: evaluating the current understanding to identify the knowledge gaps and future research priorities. Sci. Total. Environ..

[9] Sarker A (2020). A review of microplastics pollution in the soil and terrestrial ecosystems: a global and Bangladesh perspective. Sci. Total Environ..

[10] Tang KHD (2020). Effects of microplastics on agriculture: a mini-review. Asian J. Environ. Ecol..

[11] Bandmann V, Müller JD, Köhler T, Homann U (2012). Uptake of fluorescent nano beads into BY2-cells involves clathrin-dependent and clathrin-independent endocytosis. FEBS Lett..

[12] Carbery M, O’Connor W, Palanisami T (2018). Trophic transfer of microplastics and mixed contaminants in the marine food web and implications for human health. Environ. Int..

[13] Purkayastha D, Sarkar S (2022). Black soldier fly larvae for treatment and segregation of commingled municipal solid waste at different environmental conditions. J. Environ. Manag.

[14] Klammsteiner T, Turan V, Fernández-Delgado Juárez M, Oberegger S, Insam H (2020). Suitability of black soldier fly frass as soil amendment and implication for organic waste hygienization. Agronomy.

[15] Liu T (2022). Black soldier fly larvae for organic manure recycling and its potential for a circular bioeconomy: a review. Sci. Total Environ..

[16] Cho S, Kim C-H, Kim M-J, Chung H (2020). Effects of microplastics and salinity on food waste processing by black soldier fly (*Hermetia**illucens*) larvae. J. Ecol. Environ..

[17] Rondoni G, Chierici E, Agnelli A, Conti E (2021). Microplastics alter behavioural responses of an insect herbivore to a plant–soil system. Sci. Total Environ..

[18] Romano N, Fischer H (2021). Microplastics affected black soldier fly (*Hermetia illucens*) pupation and short chain fatty acids. J. Appl. Entomol..

[19] Xu C (2020). Are we underestimating the sources of microplastic pollution in terrestrial environment?. J. Haz. Mater..

[20] Ainali NM (2022). Do poly(lactic acid) microplastics instigate a threat? a perception for their dynamic towards environmental pollution and toxicity. Sci. Total Environ..

[21] Fernández-González V, Andrade JM, Ferreiro B, López-Mahía P, Muniategui-Lorenzo S (2021). Monitorization of polyamide microplastics weathering using attenuated total reflectance and microreflectance infrared spectrometry. Spectrochim. Acta Part A Mol. Biomol. Spectrosc..

[22] Sheppard DC, Tomberlin JK, Joyce JA, Kiser BC, Sumner SM (2002). Rearing methods for the black soldier fly (Diptera: Stratiomyidae). J. Med. Entomol..

[23] Mulisch, M., Welsch, U. (eds.): Romeis-Mikroskopische Technik. Springer, Berlin (2015). 10.1007/978-3-642-55190-1

[24] Lievens S (2022). Mutual influence between polyvinyl chloride (micro)plastics and black soldier fly larvae ( * Hermetia *
* illucens * L.). Sustainability.

[25] Xu Z (2023). Microplastics existence intensified bloom of antibiotic resistance in livestock feces transformed by black soldier fly. Environ. Pollut..

[26] Lievens S (2023). Ingestion and excretion dynamics of microplastics by black soldier fly larvae and correlation with mouth opening size. Sci. Rep..

[27] Heussler, C., Insam, H., Walter, A., Steiner, B.S., Steiner, F., Klammsteiner, T.: Life-history traits of black soldier fly reared on agro-industrial by-products subjected to three pre-treatments: A pilot-scale study. J. Insects Food Feed. **9**(5), 545–556 (2022). 10.3920/JIFF2022.0044

[28] Lalander C, Diener S, Zurbrügg C, Vinnerås B (2019). Effects of feedstock on larval development and process efficiency in waste treatment with black soldier fly ( * Hermetia illucens * ). J. Clean Prod..

[29] Heussler, C.D., Klammsteiner, T., Stonig, K.T., Insam, H., Schlick-Steiner, B.C., Steiner, F.M.: Decrypting the microbiota on the black soldier fly’s (*Hermetia**illucens* L., Diptera: Stratiomyidae) egg surface and their origin during development. BioRxiv (2022). 10.1101/2022.12.22.520758

[30] Qin W (2022). Effects of biochar amendment on bioconversion of soybean dregs by black soldier fly. Sci. Total Environ..

[31] Beutel, R., Pohl, H.: Insecta (*Hexapoda*). In: Westheider, W., Rieger, G. (eds.) Spezielle Zoologie. Teil 1: Einzeller und Wirbellose Tiere, pp. 634–710. Springer Spektrum Berlin, Heidelberg (2013)

[32] Bruno D (2019). The digestive system of the adult *Hermetia**illucens* (Diptera: Stratiomyidae): morphological features and functional properties. Cell Tissue Res..

[33] Rung C, Welle F, Gruner A, Springer A, Steinmetz Z, Munoz K (2023). Identification and evaluation of (non-)intentionally added substances in post-consumer recyclates and their toxicological classification. Recycling.

[34] Klammsteiner T (2020). The core gut microbiome of black soldier fly ( * Hermetia illucens * ) larvae raised on low-bioburden diets. Front. Microbiol..

[35] Klammsteiner T (2021). Impact of processed food (canteen and oil wastes) on the development of black soldier fly ( * Hermetia illucens * ) larvae and their gut microbiome functions. Front. Microbiol..

[36] LeMoine CMR, Grove HC, Smith CM, Cassone BJ (2020). A very hungry Caterpillar: polyethylene metabolism and lipid homeostasis in larvae of the greater wax moth (*Galleria**mellonella*). Environ. Sci. Technol..

[37] Yang S-S (2018). Ubiquity of polystyrene digestion and biodegradation within yellow mealworms, larvae of *Tenebrio**molitor* Linnaeus (Coleoptera: Tenebrionidae). Chemosphere.

